# Establishment and characterization of a radiation‐induced dermatitis rat model

**DOI:** 10.1111/jcmm.14174

**Published:** 2019-02-28

**Authors:** Xiaowu Sheng, Yue Zhou, Hui Wang, Yongyi Shen, Qianjin Liao, Zhen Rao, Feiyan Deng, Luyuan Xie, Chaoling Yao, Huangxing Mao, Zhiyan Liu, Mingjing Peng, Ying Long, Yong Zeng, Lei Xue, Nina Gao, Yu Kong, Xiao Zhou

**Affiliations:** ^1^ Hunan Branch Center, National Tissue Engineering Center of China, Translational Medical Center, Central Laboratory, Hunan Cancer Hospital and The Affiliated Cancer Hospital of Xiangya School of Medicine, Central South University Changsha Hunan Province China; ^2^ Department of Radiation Oncology Key Laboratory of Translational Radiation Oncology Changsha Hunan Province China; ^3^ Department of Radiation Oncology Hunan Cancer Hospital and The Affiliated Cancer Hospital of Xiangya School of Medicine, Central South University Changsha Hunan Province China; ^4^ Xiangya School of Medicine, Central South University Changsha Hunan Province China; ^5^ Nursing Department Hunan Cancer Hospital and The Affiliated Cancer Hospital of Xiangya School of Medicine Changsha Hunan Province China; ^6^ Department of Head and Neck Surgery The First People's Hospital of Changde City Changsha Hunan Province China; ^7^ University of South China Hengyang Hunan Province China; ^8^ Department of Head and Neck Surgery Hunan Cancer Hospital and The Affiliated Cancer Hospital of Xiangya School of Medicine Changsha Hunan Province China; ^9^ Pathology Department Hunan Cancer Hospital and The Affiliated Cancer Hospital of Xiangya School of Medicine Changsha Hunan Province China; ^10^ Institute of Neuroscience Chinese Academy of Sciences Shanghai China

**Keywords:** apoptosis, autophagy, fibrosis, radiation‐induced dermatitis

## Abstract

Radiation‐induced dermatitis is a common and serious side effect after radiotherapy. Current clinical treatments cannot efficiently or fully prevent the occurrence of post‐irradiation dermatitis, which remains a significant clinical problem. Resolving this challenge requires gaining a better understanding of the precise pathophysiology, which in turn requires establishment of a suitable animal model that mimics the clinical condition, and can also be used to investigate the mechanism and explore effective treatment options. In this study, a single dose of 90 Gy irradiation to rats resulted in ulceration, dermal thickening, inflammation, hair follicle loss, and sebaceous glands loss, indicating successful establishment of the model. Few hair follicle cells migrated to form epidermal cells, and both the severity of skin fibrosis and hydroxyproline levels increased with time post‐irradiation. Radiation damaged the mitochondria and induced both apoptosis and autophagy of the skin cells. Therefore, irradiation of 90 Gy can be used to successfully establish a rat model of radiation‐induced dermatitis. This model will be helpful for developing new treatments and gaining a better understanding of the pathological mechanism of radiation‐induced dermatitis. Specifically, our results suggest autophagy regulation as a potentially effective therapeutic target.

## 1 INTRODUCTION

Radiotherapy is the main treatment for malignant tumours and administered to more than 50% of cancer patients worldwide.^1^ In general, 95% of patients who undergo radiotherapy will develop a variety of serious complications, including radiation‐induced dermatitis in the form of either early or late skin reactions.^2^ Acute radiation‐induced dermatitis typically occurs within a few days or months after the beginning of irradiation, and is characterized by erythema, ulceration, pigment changes and dry or moist desquamation, whereas chronic radiation‐induced dermatitis is defined when the injury lasts a few months or years and is accompanied by delayed healing, along with irreversible and progressive fibrosis.^3^, ^4^ Currently, a variety of interventions are used to treat radiation‐induced dermatitis, such as washing, typical dermatitis agents (hyaluronic acid, corticosteroid, sucralfate cream), antioxidant agents (vitamin C and vitamin E) and wound dressing (hydrocolloid or hydrogel); however, the majority of patients with a severe form of radiation‐induced dermatitis usually requires surgical treatment.^5^, ^6^ Despite the wide array of treatment options, the curative effects remain unsatisfactory, imposing a major burden on the patients’ quality of life. Therefore, further research effort is needed to uncover the underlying mechanism of radiation‐induced dermatitis to guide development of novel therapeutic strategies. Although in vitro analyses can be informative, proper understanding of the pathological mechanisms requires establishment of a suitable animal model that can mimic the human clinical condition.

Radiation‐induced cell death is mediated by induction of cellular apoptosis, necrosis and autophagy,^7^ and thus the mechanism is directly and indirectly related to DNA damage.^8^ Ionizing radiation breaks the chemical bonds on the helical backbone, resulting in single‐strand breaks and double‐strand breaks that ultimately lead to cell death or poor DNA repair. The destruction of chemical bonds also generates reactive oxygen species (ROS), which can damage DNA and organelles, causing mitochondrial dysfunction and other cellular irregularities.^9^ These DNA breaks and ROS activate several transduction pathways such as nuclear factor‐kappa B (NF‐B) and p53.^10^ In particular, NF‐B activation can regulate genes of the B‐cell lymphoma‐2 (Bcl‐2) family that encode proteins with either pro‐apoptotic or anti‐apoptotic activity,^11^, ^12^ including the anti‐apoptotic Bcl‐2 and pro‐apoptotic Bax proteins.^13^, ^14^ Similarly, p53 activation induces irreversible G1/S or G2/M cell‐cycle arrest, senescence, and apoptosis^15^, ^16^ but can also activate the energy sensor AMPK pathway, inhibit the mammalian target of rapamycin (mTOR) pathway, and transactivate multiple genes with pro‐autophagic effects.^17^, ^18^, ^19^, ^20^ Autophagy is a cellular degradation and recycling process that has emerged as an important cytoprotective mechanism to limit ROS accumulation and prevent apoptosis.^21^ Recent studies have shown that ROS could act as cellular signalling molecules to initiate autophagosome formation and autophagic degradation,^22^ whereas autophagy can reduce oxidative damage and ROS levels through removal of protein aggregates and damaged organelles such as mitochondria.^23^ Overall, radiation‐induced autophagy may have bidirectional effects in determining the cell fate by degrading the damaged organelles to maintain cell viability or to promote excessive self‐degradation to induce cell death.^24^ Shao et al^25^ indicated that irradiation activates mTORC1 signalling in the kidneys, leading to inhibition of cellular autophagy. However, it is still unclear whether radiation activates or inhibits autophagy in radiation‐induced dermatitis.

To help resolve these questions, a rat model of radiation‐induced dermatitis was established in this study, and activation of autophagy and apoptosis was evaluated in the model rats using morphological and biochemical methods. This study will provide an experimental basis to facilitate further investigations of the pathogenesis of radiation‐induced dermatitis towards setting a new foundation for treatment.

## 2 MATERIALS AND METHODS

### 2.1 Experimental animals

Sprague‐Dawley rats weighing 220‐250 g were used in this study. All researchers that were involved in this study received animal experiments trainings from Central South University and obtained certificates. All rats had free access to food and water and were pre‐fed for 7 days to adapt to the environment. Ethics approval and consent to participate were obtained from Animal Ethics Committee of Hunan Cancer Hospital. The affiliated cancer hospital of Xiangya School of Medicine, Central South University.

### 2.2 Establishment of the radiation‐induced dermatitis rat model

The rats were irradiated following a previously reported procedure.^26^ In brief, 5% pentobarbital sodium (0.1 mL/100 g) was injected intraperitoneally for anaesthesia, and then the surface of the medial rectus femoris on the left thigh was labelled using a marker. Radiation was administered by a Nucletron Microselectron‐HDR Ir‐192 system after loading (Nucletron Company, the Netherlands), with the applicator tube fixed at the mark made on the left thigh. The dose normalization point was 0.5 cm below the source center, and the irradiation area was a 0.5‐cm radius around the marked point. The rats were randomly divided into three groups (n = 12 per group, with an equal sex ratio): control group, without irradiation; 4 weeks post‐irradiation group, exposed to a single dose of 90 Gy irradiation, and assessed after 4 weeks; 12 weeks post‐irradiation group, exposed to a single dose of 90 Gy irradiation and assessed at 12 weeks after irradiation.

### 2.3 Score evaluation

The rats were examined after irradiation by two blinded observers. The degree of skin tissue toxicity was scored according to previously reported skin score criteria for an animal model^27^, ^28^ (Table 1). Skin damage was measured for 12 weeks, and several pictures of the irradiated skin area were taken with a digital camera.

**Table 1:** Score evaluation criteria for radiation‐induced dermatitis of rat skin

Score	Observation
1.0	No effect
1.5	Minimal erythema, mild dry skin
2.0	Moderate erythema, dry skin
2.5	Marked desquamation, minimal dry crusting
3.0	Dry desquamation, minimal dry crusting
3.5	Dry desquamation, dry crusting, superficial minimal scabbing
4.0	Patchy moist desquamation, moderate scabbing
4.5	Confluent moist desquamation, ulcers, large deep scabs
5.0	Open wound, full‐thickness loss
5.5	Necrosis

### 2.4 Haematoxylin‐eosin staining

The irradiated rectus femoris skin tissues were fixed with 4% paraformaldehyde overnight, paraffin‐embedded and sliced into sections of 4 m. The sections were dewaxed with xylene, rehydrated with graded concentrations of ethanol and then stained with haematoxylin and eosin reagents (Solarbio, G1120). An inverted microscope (Zeiss, Axio Scope A1) was used for observation.

### 2.5 Masson trichrome staining

The irradiated rectus femoris skin tissues were fixed with 4% formaldehyde for 24 hours, and were then paraffin‐embedded and sliced into sections of 4 m. Masson trichrome staining (Baso, BA‐40798) was used after slice dewaxing, and the stained sections were observed under the inverted microscope (Zeiss, Axio Scope A1) All visual fields in each slice were selected, and the percentage of the collagen fibrosis area was analysed by Image‐Pro Plus 6 software (Media Cybernetics, Bethesda, MD, USA).

### 2.6 Hydroxyproline content

The hydroxyproline content in the irradiated skin tissues was used to quantify the collagen content using the hydroxyproline analysis kit (Nanjing Jiancheng Bioengineering Institute, A030‐2) according to the manufacturer's protocol, since collagen contains approximately 13.5% hydroxyproline.^29^ In brief, the skin tissues were removed of blood, rapidly frozen in liquid nitrogen and stored at −80°C. The results are reported as micrograms of hydroxyproline per gram of tissue wet weight.

### 2.7 Electron microscopy

The irradiated skin tissues were fixed with 2.5% glutaraldehyde overnight and then embedded and sliced. The ultrastructure of the skin cells and organelles was observed by Tecnai G2 Spirit and electron microscopy (FEI, Hillsboro, OR, USA). A GATAN ORIUS CCD camera (GATAN, Pleasanton, CA, USA) was used to collect images.

### 2.8 TUNEL staining

The irradiated rectus femoris skin tissues were fixed with 4% formaldehyde for 24 hours, and then paraffin‐embedded and sliced into sections of 4 m. Apoptotic cells were detected by a TUNEL assay with the in situ Cell Death Detection Kit (Roche, 11684817910) according to the manufacturer's protocol. In brief, paraffin sections were deparaffinized, permeabilized with proteinase K, incubated with a mixture of nucleotides and TdT enzyme, and then stained with DAPI. An inverted fluorescence microscope (Zeiss, Axio Scope A1) was used for observation to count TUNEL‐positive cells (green nuclei) under ×200 magnification in a blinded manner.

### 2.9 Western blot

The protein levels of Bcl‐2, Bax, p53, LC3 and Beclin‐1 in the skin tissues were examined by western blotting. The total protein was extracted from the cell lysate of the irradiated skin tissue of the rats, and 20 μg protein was loaded onto a 10% sodium dodecyl sulphate polyacrylamide gel for electrophoresis. After electrophoresis, the protein was transferred to a polyvinylidene fluoride membrane. The membrane was incubated in 5% skimmed milk for 1 hour, followed by incubation with the primary antibody overnight at 4°C. The antibodies used included polyclonal rabbit anti‐Bcl‐2 antibody (12789‐1‐AP, Proteintech, Rosemont, IL, USA; 1:2000 dilution), polyclonal rabbit anti‐Bax antibody (50599‐2‐Ig, Proteintech; 1:4000 dilution), polyclonal rabbit anti‐p53 antibody (10442‐1‐AP, Proteintech; 1:3000 dilution), polyclonal rabbit anti‐LC3B antibody (ab48394, Abcam, 1:1000 dilution), monoclonal rabbit anti‐Beclin‐1 antibody (D40C5, #3495, CST, Danvers, MA, USA; 1:1000 dilution), and monoclonal rabbit anti‐β‐actin antibody (7D2C10, 60008‐1‐Ig, Proteintech; 1:5000 dilution). Then, the membrane was incubated with the secondary antibody (sheep anti‐rabbit IgG conjugated to horseradish peroxidase; 1:3000 dilution) at room temperature for 1 hour. The membrane was washed, and the bands were visualized with enhanced chemiluminescence.

### 2.10 Immunohistochemical staining

The irradiated rectus femoris skin tissues were fixed with 4% formaldehyde for 24 hours, and then paraffin‐embedded and sliced into sections of 4 m. Sections of skin tissue were stained using polyclonal rabbit anti‐LC3B antibody (ab48394, Abcam, 1:200 dilution) and polyclonal rabbit anti‐Beclin‐1 antibody (ab62557, Abcam, Cell Signaling Technology, 1:200 dilution). Nuclei of cells were counterstained with hematoxylin, then sections were dehydrated and mounted.

### 2.11 Statistical analysis

All data are represented as mean ± standard error of the mean. The experimental data were processed by SPSS 13.0 software (SPSS, Chicago, IL, USA), and differences between groups were analysed by a *t* test; *P* < 0.05 indicated a statistically significant difference.

## 3 RESULTS

### 3.1 Radiation of 90 Gy induced acute and chronic skin reactions in rats

**Figure 1:**
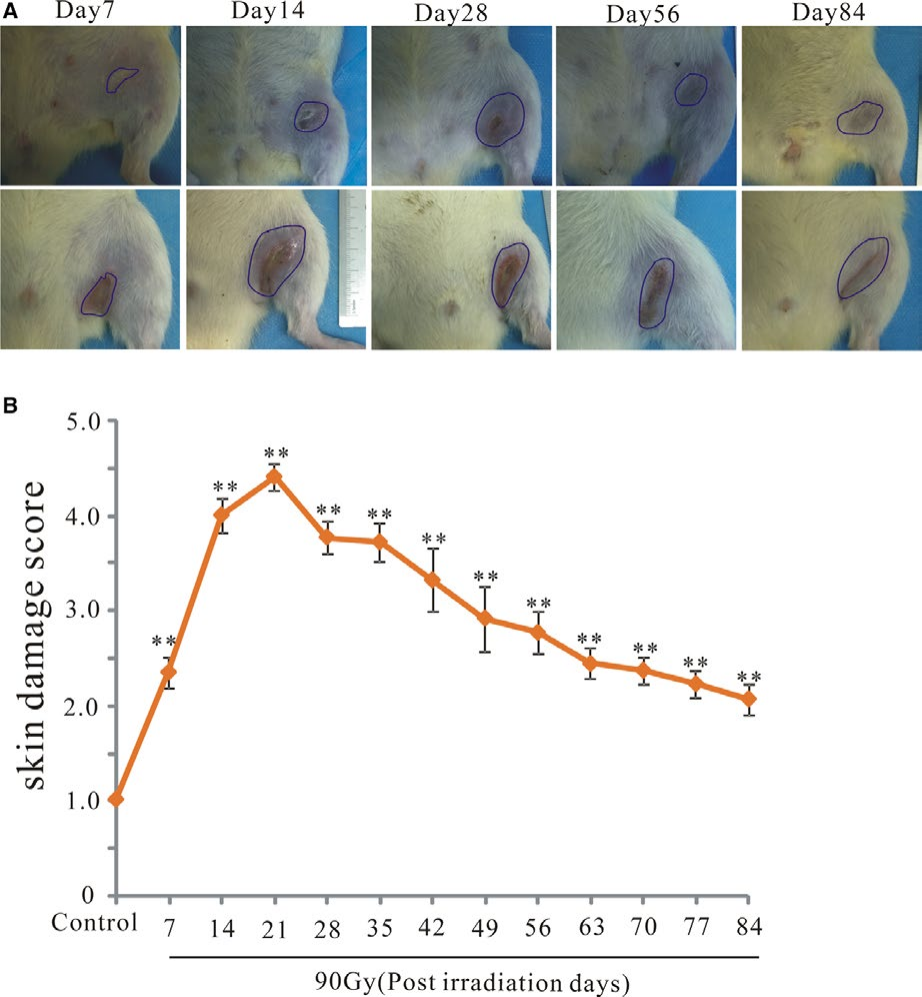
Radiation of 90 Gy induced acute and chronic skin reactions in rats. Skin injury was measured by semiquantitative scoring from 1 (no damage) to 5 (severe damage). A, Representative skin images from 90 Gy irradiated skin tissue at 7, 28, 56 and 84 d after irradiation. B, Skin samples were visually scored for radiation injury. The results are expressed as the mean ± SEM. *P < 0.05, compared with the control group

We previously demonstrated that 90 Gy irradiation induced skeletal muscle fibrosis in Sprague‐Dawley rats.^26^ Therefore, we used 90 Gy irradiation to establish a rat model of radiation‐induced dermatitis. The skin was evaluated for injury at 4 and 12 weeks following a single dose of irradiation. Visible damage was detected in the skin as of 7 days post‐irradiation. At 4 weeks after irradiation, the skin of all rats had ulcers, erythema, and evident hair loss. Most of this visible skin damage was repaired by scar tissue at 8 weeks after irradiation, and all of the damage was repaired by scar tissue by 12 weeks post‐irradiation, resulting in extensive hair loss (Figure 1A). The changes in skin injury scores during the 12 weeks following a single dose of 90 Gy irradiation are shown in Figure 1B. Thus, this model was found to be suitable for examining both acute and chronic cases of radiation‐induced dermatitis observed clinically.

### 3.2 Radiation of 90 Gy induced dermatitis in rats

**Figure 2:**
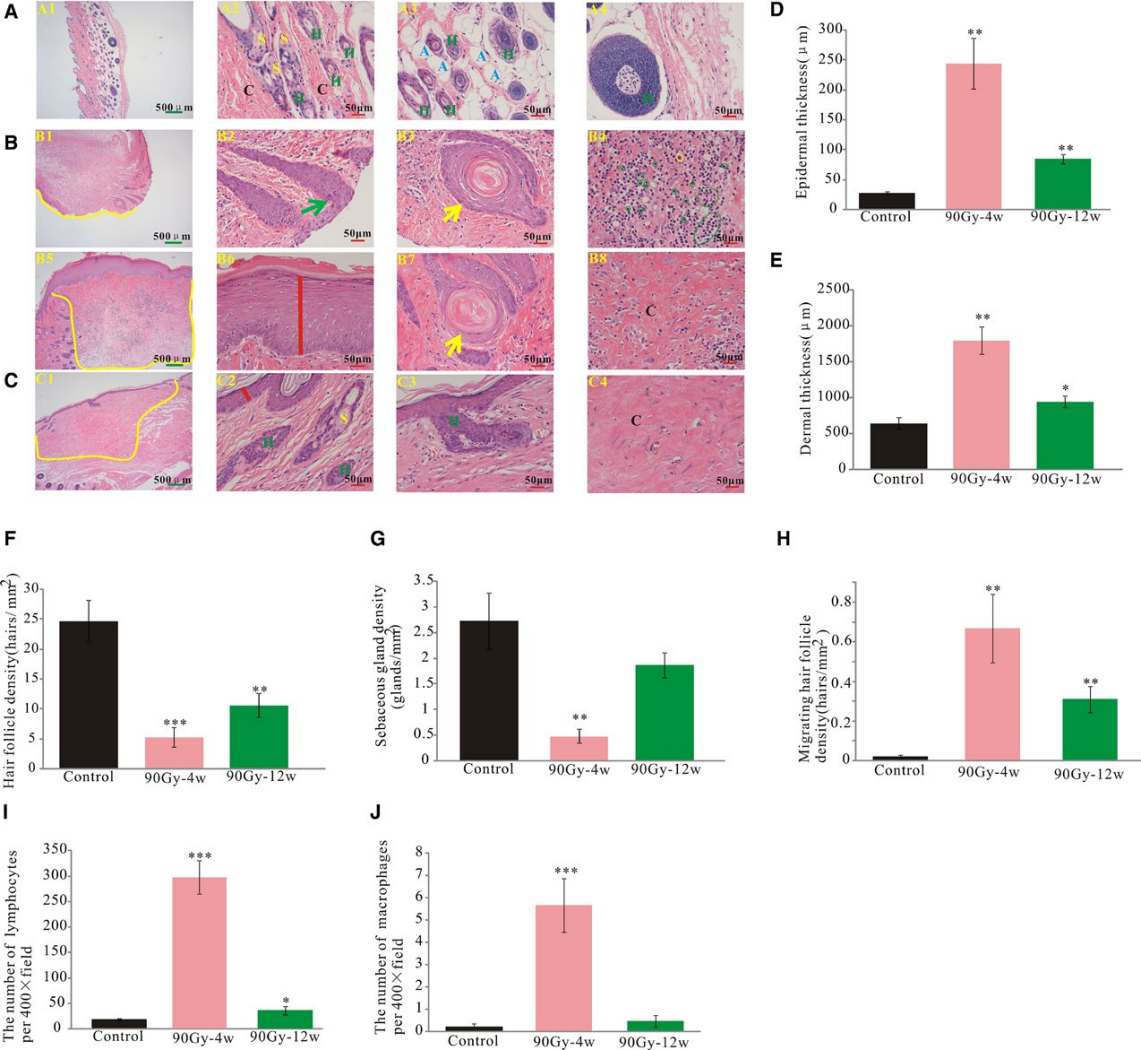
Ulceration and inflammation were formed, epidermal and dermal thickening, hair follicle and sebaceous glands loss. A, Representative haematoxylin and eosin staining of rat skin from control. B, 4 wk post‐irradiation. C, 12 wk post‐irradiation. D, The average epidermal thickness was measured for each group. E, The average dermal thickness was measured for each group. F, Hair follicle density was evaluated for each group. G, sebaceous gland density was evaluated for each group. H, Migrating hair follicle density was evaluated for each group. I, Lymphocytes number per 400× field. J, Macrophages number per 400× filed. Green arrows show the migrating hair follicle. Yellow arrows show keratinization. Yellow circles mark the infiltrated macrophages. Green circles mark the infiltrated lymphocytes. The results are expressed as the mean ± SEM. *P < 0.05, **P < 0.01, compared with control group. H: Hair follicle, S: sebaceous glands, A: adipose tissue, C: collagen

The skin from 90 Gy‐irradiated rats was obtained at 4 and 12 weeks post‐irradiation for histological analysis (Figure 2A‐C). At 4 weeks post‐irradiation, there was epidermal loss (Figure 2B1, yellow curve), migration of hair follicle cells to form epidermal cells (Figure 2B2 green arrow), increased epidermis (Figure 2B6 red line), keratinize formed (Figure 2B3 and B7 yellow arrows), macrophages (Figure 2B4 yellow circle) and lymphocytes infiltration (Figure 2B4 green circle), and skin appendage loss (Figure 2B1 and B5 yellow curve). In addition, the adipose tissue was replaced by fibrotic tissue (Figure 2B8 “C”). At 12 weeks post‐irradiation, there was clear dermal fibrosis with appendage loss (Figure 2C1 yellow curve) but no ulceration (Figure 2C). Analysis of tissue sections confirmed that the epidermis thickness significantly increased at both 4 and 12 weeks compared with that of the control group (242.83 ± 42.69 μm, n = 10, *P* < 0.01; 83.61 ± 7.81 μm, n = 10, *P* < 0.01, respectively; Figure 2D).The dermis was significantly increased at 4 weeks (1785.76 ± 185.47 μm, n = 10, *P* < 0.01) and 12 weeks (938.46 ± 84.37 μm, n = 12, *P* < 0.05) post‐irradiation compared with that of the control group (Figure 2E). Hair follicle density was significantly reduced at both 4 and 12 weeks post‐irradiation compared with that of the control (5.10 ± 1.62 hairs/mm^2^, n = 9 and 24.55 ± 3.43 hairs/mm^2^, n = 9, respectively; both *P* < 0.01, Figure 2F).The sebaceous glands density was significantly reduced compared with that of the control at 4 weeks post‐irradiation (0.46 ± 0.13 hairs/mm^2^, n = 9, *P* < 0.01), and also showed a decreased tendency at 12 weeks but did not reach statistical significance (Figure 2G). The migrating hair follicle density was significantly increased at both 4 and 12 weeks post‐irradiation compared with that of the control (0.67 ± 0.18 hairs/mm^2^, n = 9 and 0.30 ± 0.06 hairs/mm^2^, n = 9, respectively; both *P* < 0.01, Figure 2H).The number of lymphocytes per 400× filed was significantly increased at 4 weeks (295.85 ± 32.71, n = 9, *P* < 0.001, Figure 2I) and 12 weeks (35.51 ± 7.77, n = 9, *P* < 0.05, Figure 2I). The number of macrophages per 400× filed was significantly increased at 4 weeks (5.65 ± 1.20, n = 9, *P* < 0.001, Figure 2J).

### 3.3 Skin fibrosis increased with time after 90 Gy irradiation

**Figure 3:**
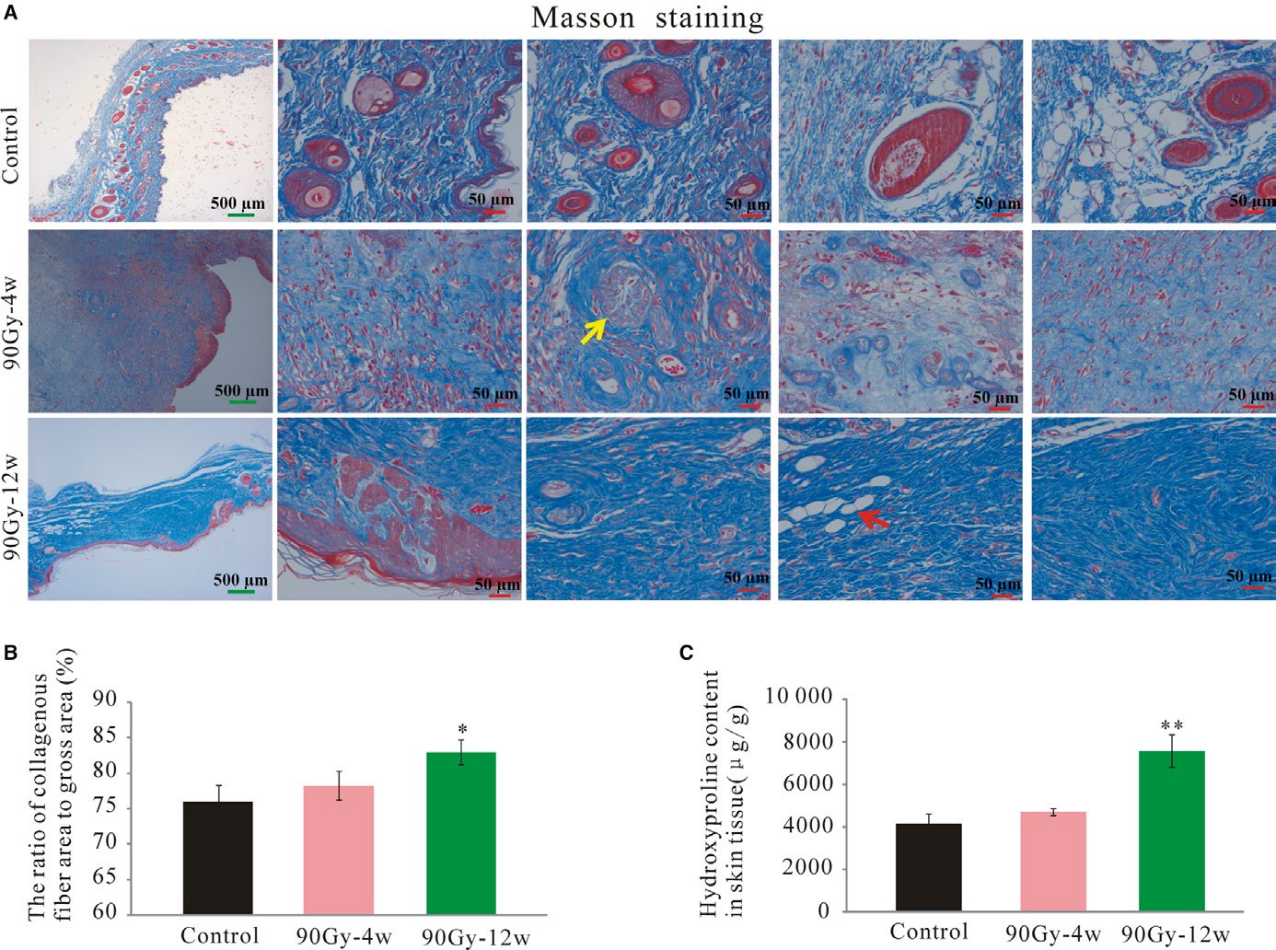
Skin fibrosis increased with time after 90 Gy irradiation. A, Representative masson staining of rat skin from control, 4 wk post‐irradiation and 12 wk post‐irradiation. B, The ratio of collagenous fiber area to gross area was measured for each group. C, Fibrosis‐related amino acid (hydroxyproline) was measured for each group. The results are expressed as the mean ± SEM. *P < 0.05, **P < 0.01, compared with control group

Masson trichrome staining showed that the skin appendages and adipose tissue were replaced by fibrotic tissue (Figure 3A yellow and red arrow, respectively) following 90 Gy irradiation. Although the skin tissue of the control group (no radiation exposure) was rich in collagen, the collagen fiber percentage decreased slightly (but not significantly) at 4 weeks post‐irradiation and then increased significantly by 12 weeks post‐irradiation (82.99 ± 1.78%, n = 8, *P* < 0.05, Figure 3B). In addition, the level of hydroxyproline, a fibrosis‐related amino acid, slightly increased at 4 weeks post‐irradiation and then significantly increased at 12 weeks compared with that of the control group (7543 ± 751.61 μg/g, n = 6, *P* < 0.01; Figure 3C).

### 3.4 Ultrastructure of irradiated skin tissue

**Figure 4:**
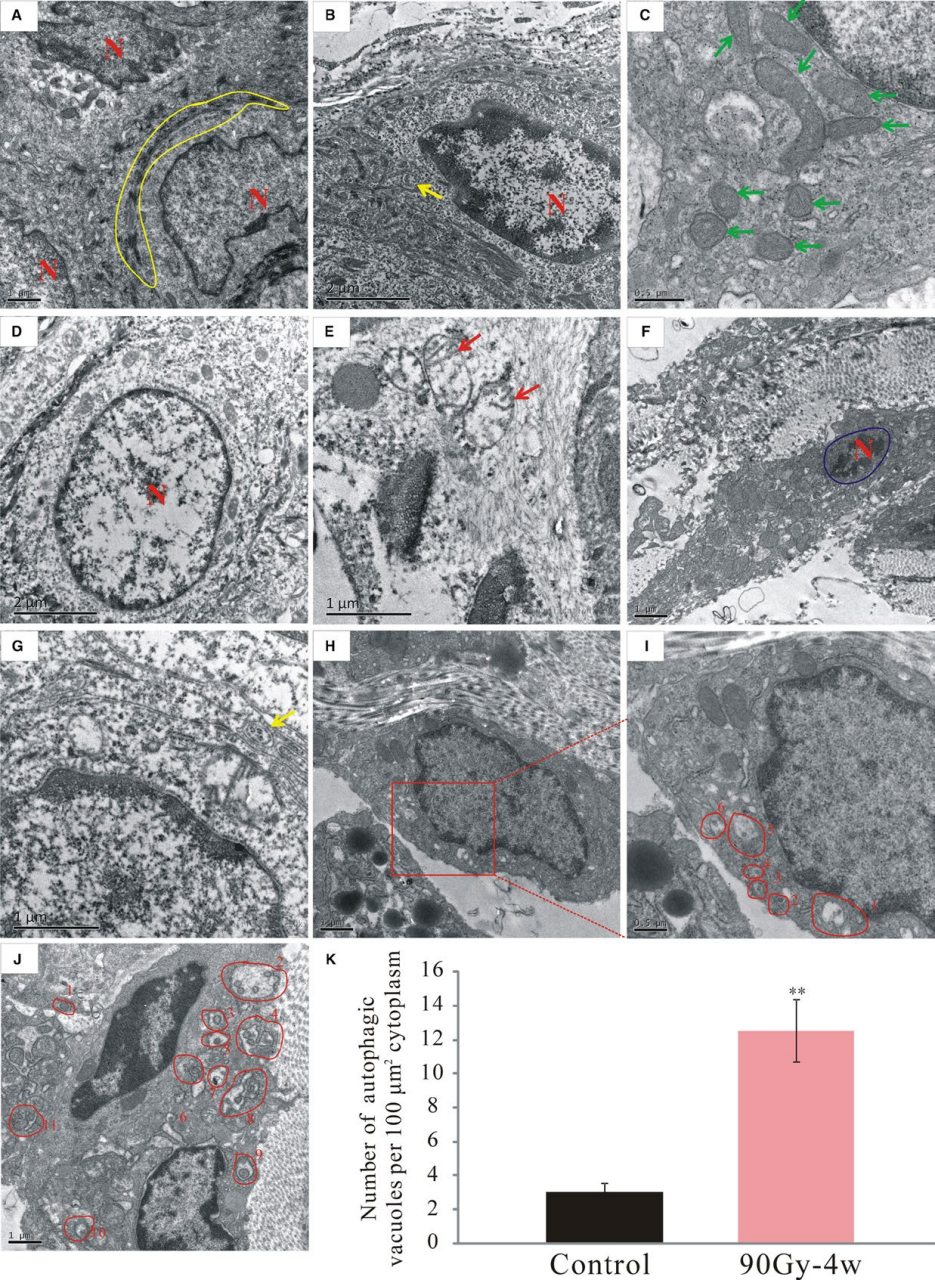
Radiation damaged the mitochondria and induced apoptosis and autophagy. The ultrastructures of the skin tissues were observed under a transmission electron microscope. (A‐C), Control group. (D‐J), Radiation‐induced dermatitis group. K, Quantification of the number of autophagic vacuoles per 100 μm^2^ cytoplasm. Green arrows show the undamaged mitochondria. Red arrows show the damaged mitochondria lost the crista structures. Yellow arrows mark the autophagic vacuoles. Yellow circles mark the desmosomes of squamous epithelial cell. Blue circles mark the apoptosis nucleus. N: nucleus of squamous epithelial cell. The results are expressed as the mean ± SEM. *P < 0.05, **P < 0.01 compared with control group

Transmission electron microscopy showed squamous cells with many desmosomes (Figure 4A, yellow circle) in the control group, with regular and normal nuclei, only a few autophagic vacuoles (Figure 4B, yellow arrow), and undamaged mitochondria (Figure 4C, green arrow). However, 4 weeks post‐irradiation, the nuclei of squamous cells were swollen (Figure 4D), and there was vacuolization of damaged mitochondria with a loss of cristae (Figure 4E, red arrow). Moreover, some nuclear shrinkage, and nuclei of irregular and small size were detected (Figure 4F, blue circle). Moreover, many more autophagic vacuoles had formed compared to the control (Figure 4G‐J, yellow arrow and red circle; Figure 4K).

### 3.5 Radiation activated apoptosis

**Figure 5:**
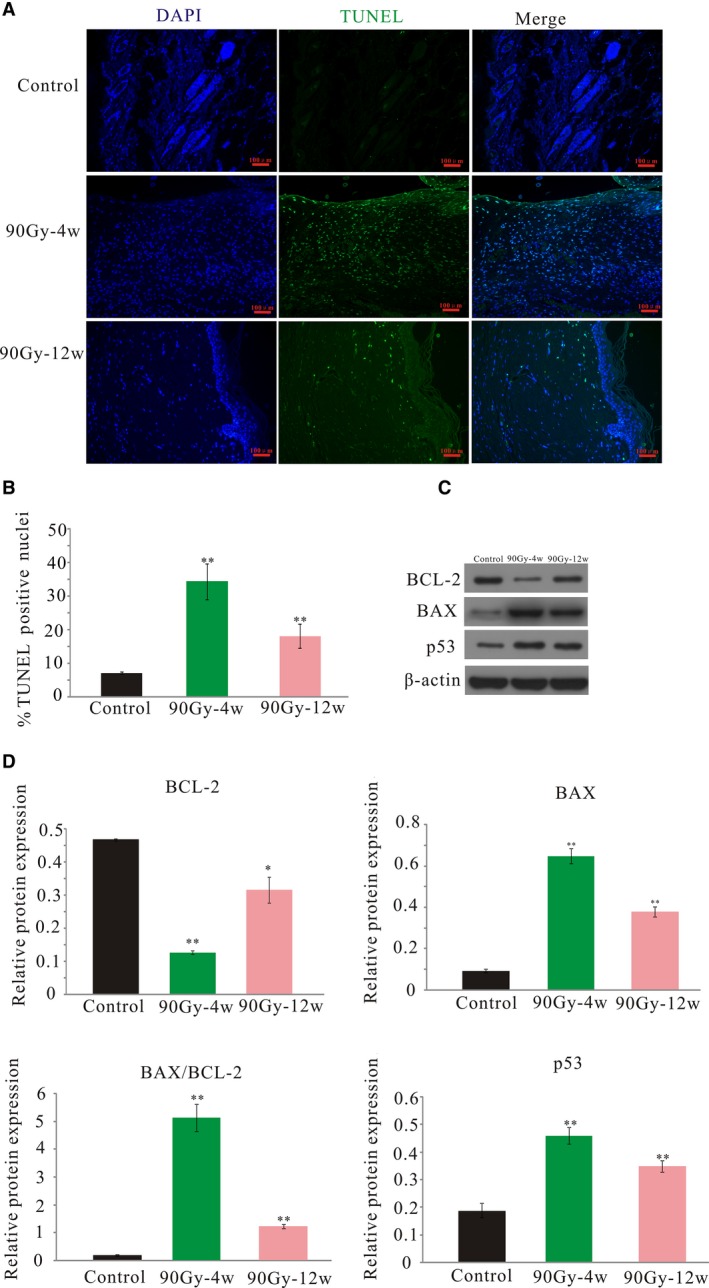
Radiation‐activated apoptosis in the skin tissue. A, Representative images of TUNEL staining. Nuclei of TUNEL‐positive cells are stained green (original magnification ×200). B, Quantificantion of TUNEL‐positive cells per high‐power field (HPF ×200). C, Representative blots of BAX, BCL‐2, and p53. D, Protein expression level analysis. The results are expressed as the mean ± SEM. *P < 0.05, **P < 0.01 compared with control group

Representative fluorescence photomicrographs of TUNEL‐positive apoptotic cells from each group are shown in Figure 5A. In contrast to the control group with only a few rare apoptotic cells, significantly more apoptotic cells were found 4 and 12 weeks after irradiation; although the numbers of apoptotic cells slightly decreased at 12 weeks, this difference from the 4‐weeks group was not statistically significant (Figure 5B). Since we previously found that irradiation‐induced apoptosis might be associated with mitochondria damage,^26^ which has been generally linked to apoptosis induction,^30^ we further analysed the expression of apoptosis‐related proteins with western blotting. Representative immunoblots are shown in Figure 5C, demonstrating that the expression level of the anti‐apoptotic protein Bcl‐2 significantly increased, whereas the levels of the pro‐apoptotic proteins Bax and p53 markedly decreased at both 4 and 12 weeks after irradiation. Correspondingly, the Bax/Bcl‐2 ratio also significantly increased at 4 and 12 weeks post‐irradiation. Collectively, these findings suggested that radiation triggered apoptosis in the skin cells.

### 3.6 Radiation activated autophagy in the skin tissue

**Figure 6:**
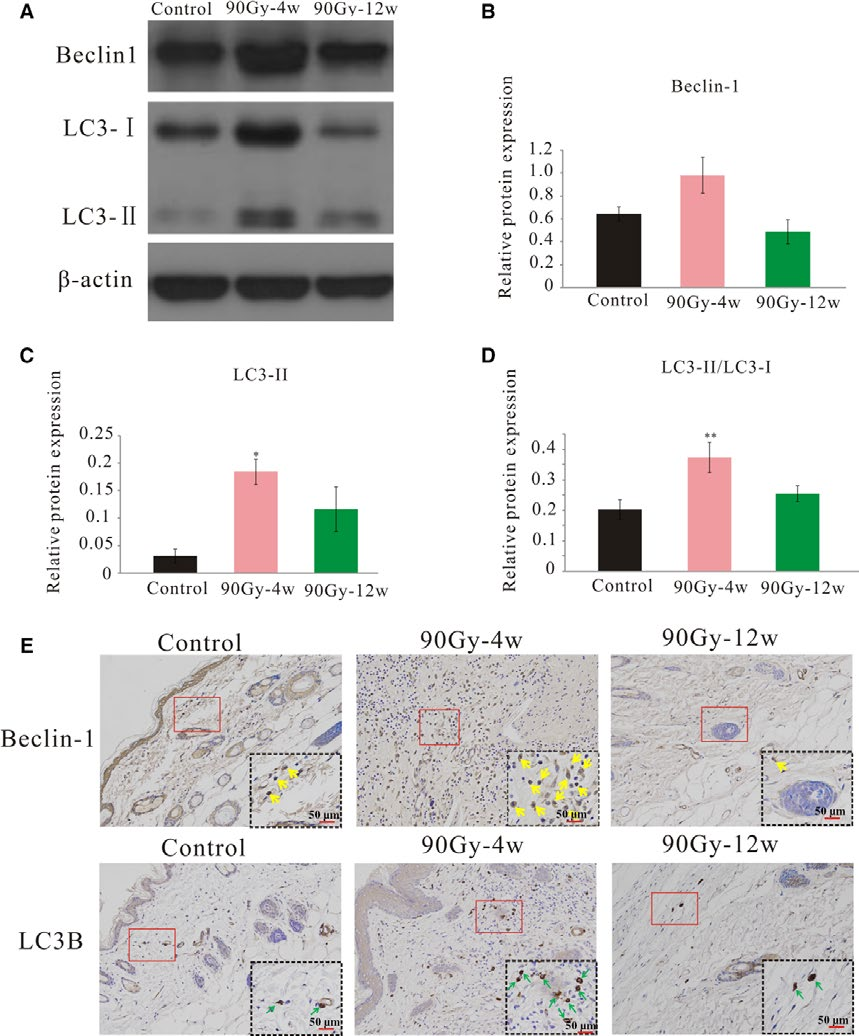
Radiation activated autophagy in the skin tissue. A, Representative blots of LC3I/II and Beclin‐1 detected by western blot, β‐actin was used as a loading control. B‐D. Protein expression level analysis. E, Representative immunohistochemistry images were shown with staining for LC3B and Beclin‐1. Yellow arrows indicated Beclin‐1 positive expression. Green arrows indicated LC3B positive expression. The results are expressed as the mean ± SEM. *P < 0.05, **P < 0.01 compared with control group

In line with the electron microscopy observations of increased autophagosomes and autophagic vacuoles in the irradiated skin tissue, the levels of the autophagy‐related proteins LC3‐II and LC3‐II/LC3‐I significantly increased, and the level of Beclin‐1 also tended towards a slight increase, but the difference did not reach statistical significance from the control after 4 weeks. The expression levels of LC3‐II and LC3‐II/LC3‐I were still slightly increased at 12 weeks, but the difference was no longer statistically significant (Figure 6A‐D). Representive immunohistochemistry staining images were shown in Figure 6E. Beclin‐1 and LC3B expression were found in the cytosol of the cells and were higher in 4 weeks post‐irradiation compared with the control. Overall, these findings indicated that irradiation could induce autophagy.

## 4 DISCUSSION

Radiation‐induced dermatitis is a common and serious side effect in patients with breast cancer or head and neck cancer who receive radiotherapy^31^, ^32^; however, there is currently no effective treatment, thus remaining a significant clinical concern that further negatively affects the patient's quality of life.^3^, ^33^ Towards promoting research into the pathophysiology of this response, we successfully established radiation‐induced acute and chronic dermatitis rat models using 90 Gy of radiation exposure to the skin.

The severity of radiotherapy‐induced tissue damage increases in proportion to the total dose and fraction size, but the use of multiple and smaller radiation fractions can help to prevent chronic damage.^34^, ^35^ Jourdal et al^36^ developed a rat model of skin injury due to the combined effects of radiation (single dose of 10‐40 Gy) and a wound, and irradiation delayed wound healing in a dose‐dependent manner. Takikawa et al^37^ exposed rats to single doses of 10, 15, 20, and 30 Gy, respectively, and reported that the damage induced to rats following 30 Gy irradiation did not heal within 6 months, but the skin wound and skin score were not evaluated long term in the study. Rodgers et al^38^ used low‐penetrating X‐rays with different single radiation doses to generate a guinea pig cutaneous radiation‐induced injury model; skin damage was time‐ and radiation dose‐dependent, but the skin wounds were not monitored beyond 1 month, even if they had not healed. Similarly, Won et al^39^ developed a porcine skin injury model with a single fraction of 15, 30, 50, or 75 Gy and found that the skin injury completely healed following 15 Gy irradiation, but complete recovery was not possible in 12 weeks with a radiation dose greater than 50 Gy. Because four different radiation doses were delivered to different sections of the same mini pig, there may have been interactions between these doses, and thus the actual dose delivered to each specific region was unclear. In this study, we delivered a single dose of radiation to one section in each experimental rat to minimize effects from other parts of the body on the radiation response. We monitored the skin response in the rats for 12 weeks until the wound healed with scar formation. The data demonstrated that the skin reaction was similar to that observed in patients at different times.

The skin is one of the most sensitive tissues to radiation.^40^ The healing of skin damage largely depends on cell proliferation, along with stem cell activation, differentiation, and migration. Several types of stem cells have been identified in the skin, including epithelial, epidermal, and hair follicle stem cells. Epithelial stem cells can migrate to the epidermis in response to a wound to promote re‐epithelialization,^41^, ^42^ and a lineage tracing study showed that follicular cells can be converted to epidermal cells.^43^ Hair follicle stem cells were also shown to mobilize to the epidermis after wounding.^44^ However, our results showed that few hair follicle cells migrated to form epidermal cells at 4 weeks post‐irradiation, indicating that few hair follicle stem cells were retained in the skin tissue that differentiated into epidermal cells, and there was no obvious hair follicle and sebaceous regeneration at 12 weeks post‐irradiation. This suggests that 90 Gy irradiation induced massive stem cell loss.

Mitochondria are the energy‐producing organelles and the main cellular source of ROS,^45^ which are important triggers of apoptosis and autophagy. Mitochondrial dysfunction has been observed in a variety of radiation‐induced diseases such as injury to the skeletal muscle and lacrimal glands and brain damage.^26^, ^46^, ^47^ The damaged mitochondria release cytochrome‐c to the cytoplasm and consequently induce apoptosis.^48^, ^49^ The structural and cristae remodeling of mitochondria are associated with apoptosis.^50^, ^51^ Mitochondrial dysfunction can also cause an energy imbalance, which generates a huge amount of ROS to induce AMPK and apoptosis signal regulating kinase/c‐Jun *N*‐terminal kinase (JNK) pathway activation, which indirectly activate autophagy.^52^, ^53^, ^54^ In this study, the ultrastructural observations indicated that radiation induced severe mitochondrial damage, apoptosis and autophagy.

Radiation‐induced autophagy exerts a cytoprotective (radioresistance) or cytotoxic (radiosensitivity) effect on irradiated cells,^55^, ^56^ the fate of which is dependent on a fine balance between autophagy and apoptosis.^57^ Radiation‐induced autophagy prevents salivary gland injury post‐irradiation, and autophagy‐deficient mice display increased radiosensitivity.^58^ Our data showed that although autophagy was activated by 4 weeks after irradiation, it was not sufficient to counter radiation‐induced apoptosis. However, by 12 weeks post‐irradiation, the autophagy level decreased to a level similar to that of the control, but the ratio of apoptotic cells was much higher than that of the control group. This suggested that continuously activated apoptosis hinders the recovery from radiation‐induced dermatitis. Thus, agents or treatments that effectively regulate autophagy could help to protect cells from apoptosis, thereby preventing or alleviating radiation‐induced dermatitis. Various signaling pathways are associated with radiation‐induced autophagy induction. Ataxia‐telangiectasia mutated (ATM) is a primary sensor of DNA damage and can be activated post‐irradiation, which could promote radiation‐induced autophagy by triggering phosphorylation of its downstream targets, such as p53,^59^ mitogen‐activated protein kinase 14 (MAPK14), Forkhead box O3 (FOXO3a), mTOR and Beclin‐1/PI3KII.^60^ Furthermore, miR‐30a was shown to negatively regulate Beclin‐1 expression, resulting in decreased autophagic activity.^61^ In addition, miR‐26b reduced radiation‐induced autophagy by directly downregulating DNA damage‐regulated autophagy modulator1 (DRAM1) expression.^62^ However, the detailed underlying mechanisms of radiation‐induced autophagy are not completely elucidated. In the future, we will also analyse the underlying mechanism of radiation‐induced autophagy in radiation‐induced dermatitis.

In summary, we have established a radiation‐induced dermatitis rat model with 90 Gy irradiation, which showed a time‐dependent increase in the severity of skin fibrosis, matching well with the clinical condition. Moreover, radiation damaged the mitochondria and induced apoptosis and autophagy, indicating that apoptosis and autophagy are good therapeutic targets for preventing the development of radiation‐induced dermatitis.

## CONFLICT OF INTEREST

The authors declare no conflict of interest.
